# Impact of Dexamethasone Preconditioning on Prevention of Development of Cognitive Impairment following Acute Inflammation

**DOI:** 10.1155/2022/6064007

**Published:** 2022-06-14

**Authors:** Wenjie Cheng, Yushan Song, Yanfang Liu, Xiaohua Sun, Wanlu Ren

**Affiliations:** ^1^Department of Anesthesiology, Tianjin Hospital, Tianjin 300211, China; ^2^Jinnan District Center for Disease Control and Prevention, Tianjin 300300, China

## Abstract

To assess the preventive role of dexamethasone (Dex) in the development of neuroinflammation and concomitant neurocognitive disorders following acute inflammation. C57BL6 mice were fallen into the sham group, ischemia-reperfusion (*I*/*R*) group, and I/*R* + Dex group randomly. In the end, behavioral alterations were assessed with the Morris water maze (MWM) test, passive avoidance test (PAT), and open field test (OFT). The serum levels of IL-1*β* and TNF-*α* were detected by ELISA. Immunofluorescence was adopted to observe the NF-kB expression in the hippocampus. In addition, TLR4, NF-kB, CD68, and CD206 were examined by Western blot. The cognitive ability of mice can be impaired by tourniquet-induced acute inflammation, and these changes were prevented by Dex. Compared to the *I*/*R* group, Dex pretreatment could decrease levels of IL-1*β* and TNF-*α* proteins in serum. Besides, Dex preconditioning significantly decreased the utterance of NF-kB immunoreactive cells and TLR4, NF-kB, and CD68 overexpression in the hippocampus. Dex partly through inhibiting microglia transformation to the M1 polarization state and inactivating the TLR4/NF-kB pathway attenuates the cognitive disorders in mice.

## 1. Introduction

As an ordinary postoperative complication in geriatric patients [1], perioperative neurocognitive disorders (PNDs) are associated with higher healthcare costs, long-term disability, and even increased mortality [2]. Although the pathogenic mechanisms underlying PNDs remain elusive, the release of systemic acute inflammation and proinflammatory cytokines made after the operation is considered a critical factor in its development [3].

As the resident macrophages in the brain, microglia exert a significant effect on the inflammatory response [4]. Microglia activation can be divided into the proinflammatory M1 polarization state and the anti-inflammatory M2 stimulation status, which are related to the delivery of proinflammatory and anti-inflammatory cytokines, respectively [5, 6]. Several types of research have shown that proinflammatory cytokines including tumor necrosis element-*α* (TNF-*α*) from microglia within the hippocampus exert a pathogenic effect on cognitive disorders [7]. So far, to our knowledge, few studies report on microglial polarization on cognitive function in vivo.

In vascular or orthopedic operations, tourniquet arrangement is a universal technology to build bloodless running areas [8]. Nevertheless, tourniquet results in obvious acute limb ischemia-reperfusion (*I*/*R*) damage, with characteristics such as tissue edema, systemic inflammation, microvascular perfusion deficits [9], muscle necrosis, and even remote various organ injury [10], and these complications are risk elements for disorders of the CNS. However, to date, few papers have concentrated on the association between tourniquet use and cognitive role.

Lots of research studies have demonstrated that perioperative dexamethasone (Dex) administration may improve postoperative cognitive function [11]. Besides, Dex protectively influences *I*/*R* injury in various organs through antioxidant and anti-inflammatory properties [12, 13]. Past research displayed that the attenuated inflammation effect was related to inactivating the TLR-4/NF-kB path [14]. On basis of these outcomes, it was hypothesized that Dex through suppression of TLR-4/NF-kB pathway confers neuroprotection.

The current research investigated if tourniquet-induced acute limb ischemia-reperfusion could cause cognitive impairment and observed the role of Dex in cognitive function after tourniquet use. For exploring the potential mechanisms, the utterance of labels of microglial polarization and inflammatory cytokines in the hippocampus were investigated. Besides, we evaluated the path activation.

## 2. Materials and Methods

Experimental procedure and animal of tourniquet-stimulated acute hind limb I/R damage allowed by the Animal Care and Application Committee of the Tianjin Medical University, animal experiments were made. In all experiments, two sets of C57BL6 (Beijing Vital River Laboratory Animal Technology Co., Ltd, Beijing, China) male mice received 3-hour unilateral hind limb tourniquet ischemia, next to 24-hour reperfusion (*I*/*R*) as previously illustrated [8]. Briefly, to produce acute hind limb ischemia, the left proximal hind limb adopted a modified orthodontic rubber band as a tourniquet, then reperfusion was started by cutting the rubber band. The mice were kept in normalized laboratory surroundings (12 h illumination/12 h dim cycle with lights on at 07 : 30 am, relative humidity: 45 ± 10%, temperature:20 ± 2°C).

For the formal experiments, mice were placed into sham group, *I*/*R* group, and *I*/*R* + Dex group (*n* = 30 mice/group) randomly, and anesthetized by intraperitoneally injecting 80 mg/kg pentobarbital sodium (Aladdin, Shanghai, China). Dex (Beijing Baiaolaibo Technology Co., Ltd, Beijing, China) was diluted in 0.9% NaCl. The mice in the *I*/*R* group and *I*/*R* + Dex group received a normal saline solution or 1 mg/kg Dex intraperitoneally at 30 min before the ischemia, respectively. For the sham group, the same procedure as the *I*/*R* group except for applied orthodontic rubber band in animals. Besides, normal saline solution or Dex was intraperitoneally administered at the beginning of reperfusion. All terminal tests were made on mice after 24-hour reperfusion. The whole experiment lasted 27 hours.

### 2.1. Morris Water Maze Test

Spatial study and memory were evaluated by performing the Morris water maze (MWM) test as previously illustrated [15]. A circular pool of 120 cm in diameter and 50 cm deep was full of water until submerging a platform in the pool was 1 cm below the surface. The temperature of the water was kept at 22 ± 1°C. The swimming activity of the mice was monitored and recorded with a video analysis system (Shanghai Xinchang Information Technology, Shanghai, China). During the training trials, the mice must apply the visual tips to seek the platform hidden. For each trial, if a mouse fails to seek the platform within 90 s, it would be led to the platform and stay there for 30 s at the end. The mice received training four times a day for 5 days. The probe test was made on the sixth day after removing the platform from the pool. It was allowed for every mouse to navigate for 60 s.

### 2.2. Passive Avoidance Test

A passive avoidance study in mice [16] was evaluated with the passive avoidance test (PAT). The PAT equipment was fallen into two divisions by a retractable door: a bright and dark division. During the first designated training day, all mice were put in the bright division, and the door was opened 10 s later. Upon mice fully into the dark division, an electric shock (0.6 mA, 2 s) was received. Every mouse repeated the actions. While recording the time that the mouse invests in entering the dark compartment, a retention test was performed again 24 h later. The potential factors to re-enter the dark division and the number of electrical shocks were recorded within 5 min.

### 2.3. Open Field Test

Anxiety-like actions, spontaneous activity, and emotional variations in the animals [17] were assessed with the OFT. While being put in an open area first, mice always stay along the margin of the device due to fear. Hence, anxiety-like actions could be evaluated on the basis of the degree of exploratory action funds. The open area in the current research was made of a rectangular stage (278 × 236 mm), enclosed by a white wall 30 cm high. A single mouse in the middle was placed gently to initiate the test so that the animal could move freely for 5 min during the recording using a video analysis system (Shanghai Xinchang Information Technology, Shanghai, China).

### 2.4. ELISA of Serum IL-1*β* and TNF-*α*

After 24 hours of reperfusion, all the animals were killed, and blood samples were collected. Isolated by centrifugation, the plasma was stored at −80°C for deeper discussion. The serum degrees of IL-1*β* and TNF-*α* were investigated by enzyme associated immunosorbent assay tool on basis of the producer's guidance. We warmed the samples to room temperature and calculated the expression of IL-1*β* and TNF-*α* in serum using corresponding ELISA kits (Suzhou Calvin Biotechnology, Jiangsu, China) following standard procedures. Briefly, the standard and serum to be measured were added to the corresponding wells and then incubated using a detection antibody labeled with horseradish peroxidase. The ELISA plate was sealed with a sealing film and placed in a 36 ± 2°C water bath for 65 min. The reaction solution in the plate was removed by a pipette, and the ELISA plate was dried using filter paper after washing, followed by incubation with the corresponding substrate in a cell incubator at 37°C for 15 min. Finally, the 50 *μ*L termination solution stopped the reaction. OD values were obtained using a microplate reader (Bio-Rad, Hercules, CA, USA) at a wavelength of 450 nm.

### 2.5. Immunofluorescence Staining

Animals were killed by cervical decapitation after 24 hours of reperfusion. The brains were quickly removed and perfused with 4% paraformaldehyde (PFA) in 0.1 M sodium phosphate buffer, Ph = 7.4. Then, the overnight incubation of cerebral tissues was made in fixatives, followed by transfer to a 30% sucrose solution. Cut sequentially with a cryostat microtome (Leica, CM1850), TritonX-100 (0.3% in TBST) was adopted to permeabilize 10 *µ*m thick sections at room temperature. After 1-hour blocking in 1% bovine serum albumin, the incubation of sections was done with a 1 : 200 dilution primary antibody of rabbit antirat nuclear element kappa B (NF-kB, Abcam, Cambridge, UK) overnight at 4°C. The incubation of brain sections was made with 1 : 200 dilution of goat antirabbit secondary antibody conjugated to Cy2 (Abcam, Cambridge, UK) for 2 hours, followed by a 10-minute treatment with DAPI staining solution at room temperature the next day. Fluorescent microscopy (Olympus, BX60, Japan) was adopted to visualize immunostained sections. The capture of digital images selected for analysis was made randomly from 5 nonoverlapping fields per animal in the hippocampus subregions.

### 2.6. Protein Expression of TLR4, NF-kB, CD68, and CD206 in the Hippocampus

After 24 hours of reperfusion, hippocampus tissues from six sham groups, six *I*/*R* groups, and six Dex group mice were quickly gathered and kept at −80°C until analysis. After dissecting and splitting tissues with RIPA Lysis Buffer, the 20-minute centrifugal of tissue homogenates was made at 12,000 g and 4°C. A bicinchoninic acid protein analysis tool was adopted to decide the total protein concentration in the centrifuged supernatants. The same volume of the loading buffer was mixed with protein samples, boiled for 10 min at 95°C, and isolated with 10% sodium dodecyl sulfate-polyacrylamide gel electrophoresis. The transfer of the proteins of these samples onto a polyvinyl difluoride (PVDF) membrane was made at 200 mA for 3 hours, followed by a 1-hour blocking of the membrane with 5% nonfat milk. After being cut into strips based on molecular weights, the overnight probe of membranes was made with rabbit anti-NF-kB antibody, rabbit anti-TLR4 antibody, rabbit anti-CD206 antibody, and rabbit anti-CD68 antibody (all CST, Danvers, MA, USA) at 4°C. Washed with tris buffered saline with Tween (TBST), membranes were incubated with HRP-conjugated goat antirabbit IgG for 2 h at room temperature. *β*-Actin served as the control. In the end, TBST was adopted to wash the PVDF membrane three times, for 15 min each. A strengthened chemiluminescence substrate was adopted to visualize specific immunoreactivity, and Image J was employed to scan the specific bands for analysis.

### 2.7. Statistical Analysis

SPSS20.0 was employed to make statistical discussion. Data were shown as mean ± standard deviation (SD). Kolmogorov–Smirnov test and equal variance with Levene′s test were adopted to confirm the normal distribution of data. For multigroup comparison, statistical significance was determined with a one-way analysis of variance with the Bonferroni post hoc test. Statistical significance was *P* < 0.05.

## 3. Results

### 3.1. Spatial Learning and Memory of Mice in Different Groups

To elucidate the roles of Dex in tourniquet-induced cognitive impairment, MWM tests, PAT, and OFT were conducted.

**Figure 1:**
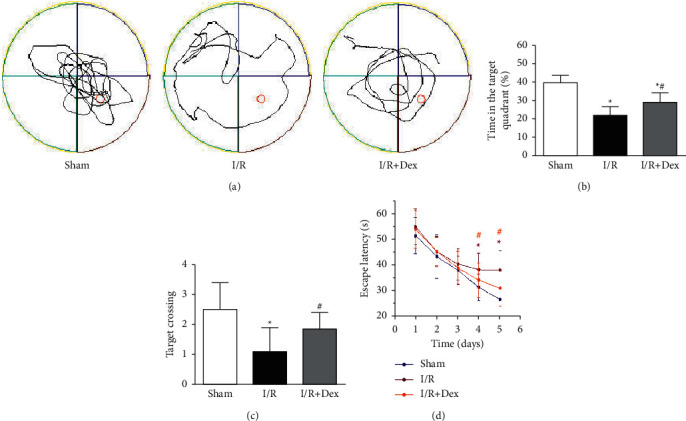
Spatial memory and tracks of mice in the Morris water maze test. (a) Typical swimming routes of mice in varied groups. (b) A quantitative exploration of the time in the target quadrant in varied groups. (c) A quantitative exploration of the number of platform crossings in varied groups. (d) A quantitative exploration of the time of escape latency for finding the platform in varied groups. Sham: control group; I/R: ischemia-reperfusion group; *I*/*R* + Dex: dexamethasone group. Data are displayed as mean ± SD (*n* = 12 in every group); ^*∗*^*P* < 0.05 vs. sham; ^*#*^*P* < 0.05 vs. I/R.

In the MWM test, according to Figure 1, the escape latency of all mice displayed a remarkable decrease over the training day. By comparing with the sham group, the escape latency was significantly longer in the *I*/*R* group on day 4 and day 5 (*P* < 0.05). Dex preconditioning noticeably shorter the latency in comparison to the *I*/*R* group (*P* < 0.05). Furthermore, the number of platform crossings was greatly reduced in the *I*/*R* group compared to the sham group (*P* < 0.05), this role was improved by Dex. According to the outcomes of the probe trial, tourniquet-induced acute limb ischemia-reperfusion was adopted to impair the learning capacity of mice, and these changes were significantly prevented by Dex preconditioning.

**Figure 2:**
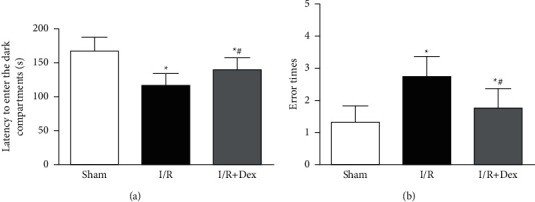
Response and memory of mice in the passive avoidance test (PAT). (a) Quantitative analysis of the latency to enter the dark division in varied groups. (b) Quantitative analysis of the mistake times to enter the dark compartment in varied groups. Sham: control group; I/R: ischemia-reperfusion group; *I*/*R* + Dex: dexamethasone group. Data are displayed as mean ± SD (*n* = 12 in every group); ^*∗*^*P* < 0.05 vs. sham; ^*#*^*P* < 0.05 vs. I/R.

In the PAT, as shown in Figure 2, it was observed that the *I*/*R* group displayed a reduced latency and more mistakes timed to enter the dark division, and these effects were greatly prevented by Dex (*P* < 0.05). The PAT outcomes displayed that tourniquet-induced acute limb ischemia-reperfusion could impair passive avoidance study in mice. These changes, however, were significantly prevented by Dex preconditioning.

**Figure 3:**
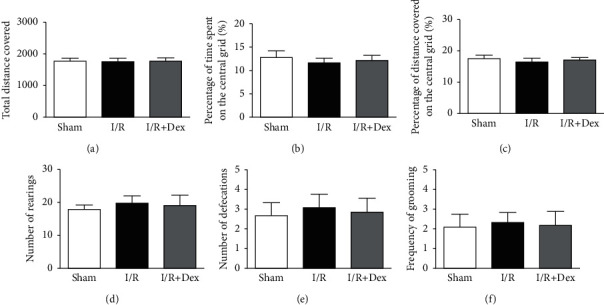
Spontaneous activities of mice in the open field test (OFT). (a) Quantitative analysis of the total distance covered in varied groups. (b) Quantitative analysis of the percentage of time spent on the central grid in varied groups. (c) Quantitative analysis of the percentage of distance covered on the central grid in varied groups. (d) Quantitative analysis of the number of rearings in varied groups. (e) Quantitative analysis of the number of defecations in varied groups. (f) Quantitative analysis of the frequency of grooming in varied groups. No great diversities were found between varied groups. Sham: control group; I/R: ischemia-reperfusion group; *I*/*R* + Dex: dexamethasone group. Data are expressed as mean ± SD (*n* = 12 in each group).

In the OFT, according to Figure 3, the actions of mice (total distance covered, percentage of distance covered on the central grid, percentage of time spent on the central grid, number of defecations and rearings, and frequency of grooming) displayed no great diversities among all groups. The action performance showed that the activity of the mice was not influenced after acute limb ischemia-reperfusion.

### 3.2. The Levels of IL-1*β* and TNF-*α* in Different Groups

**Figure 4:**
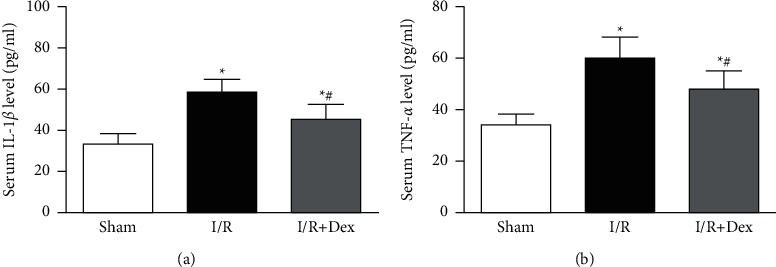
The concentration of IL-1*β* and TNF-a in all experimental groups. (a) Serum IL-1*β* level of mice in varied groups. (b) Serum TNF-a level of mice in varied groups. Sham: control group; *I*/*R*: ischemia-reperfusion group; *I*/*R* + Dex: dexamethasone group. Data are expressed as mean ± SD (*n* = 6 in each group) ^*∗*^*P* < 0.05 vs. sham; ^*#*^*P* < 0.05 vs. I/R.

According to Figure 4, by comparing with the sham group, the extents of IL-1*β* and TNF-*α* were greatly higher in the *I*/*R* group (*P* < 0.05). Dex preconditioning greatly reduced the expression of IL-1*β* and TNF-*α* proteins in serum by comparing with that in the I/R group (*P* < 0.05).

### 3.3. NF-kB Expression in the Hippocampus in Different Groups

**Figure 5:**
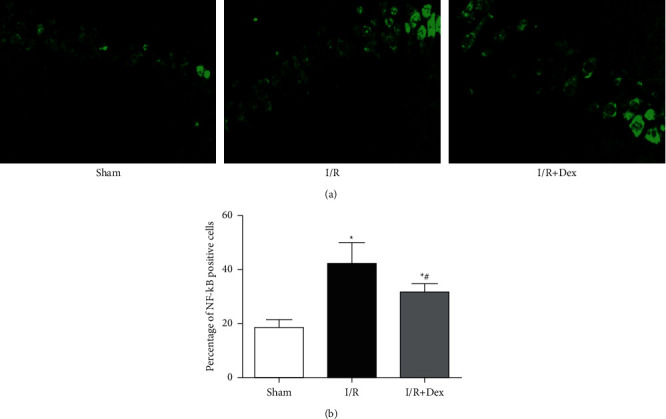
NF-kB expression in the hippocampus in different groups. (400×) (a) NF-kB immunoreactive cells in the hippocampus in different groups. (b) Quantitative analysis of the NF-kB expression in the hippocampus in different groups. Sham: control group; I/R: ischemia-reperfusion group; *I*/*R* + Dex: dexamethasone group. Data are expressed as mean ± SD (*n* = 6 in each group) ^*∗*^*P* < 0.05 vs. sham; ^*#*^*P* < 0.05 vs. I/R.

According to Figure 5, by comparing with the sham group, more NF-kB immunoreactive (IR) cells in the hippocampus were expressed in the *I*/*R* group (*P* < 0.05). Dex preconditioning greatly decreased the utterance of NF-kB IR cells in the hippocampus compared to that in the *I*/*R* group (*P* < 0.05).

### 3.4. Expression of TLR4, NF-kB, CD68, and CD206 in the Hippocampus in Different Groups

**Figure 6:**
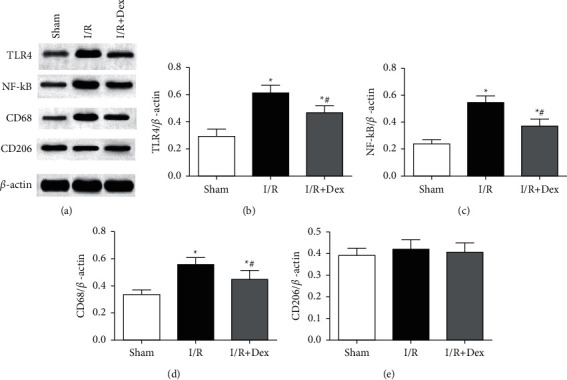
Utterance of TLR4, NF-kB, CD68, and CD206 in hippocampus examined by Western blot. (a) Representative band images of Western blot. (b) (c), (d), and (e) Summary information for the utterance of TLR4, NF-kB, CD68, and CD206 in the hippocampus. Sham: control group; I/R: ischemia-reperfusion group; *I*/*R* + Dex: dexamethasone group. Data are expressed as mean ± SD (*n* = 6 in each group) ^*∗*^*P* < 0.05 vs. sham; ^*#*^*P* < 0.05 vs. *I*/*R*.

As shown in Figure 6, the utterance of TLR4, NF-kB, and CD68 was kept at a low level in the sham group; however, these proteins levels were greatly increased in the hippocampus in the *I*/*R* group (*P* < 0.05). Dex pretreatment could observably reduce TLR4, NF-kB, and CD68 overexpression compared to that in the *I*/*R* group (*P* < 0.05). For CD206, there was no great diversity in the hippocampus between the three groups.

## 4. Discussion

The study demonstrates that tourniquet-stimulated acute limb ischemia-reperfusion can impair the study capacity of mice, and these changes were significantly prevented by Dex preconditioning. Specifically, we found decreased extents of IL-1*β* and TNF-*α* proteins in the serum of Dex pretreated mice compared to untreated. Besides, Dex pretreatment could observably decrease TLR4, NF-kB, and CD68 overexpression in the hippocampus when compared with that in the *I*/*R* group. These outcomes show that the mechanisms underlying the benefits were that Dex pretreatment partly through inhibiting microglia to transform into the proinflammatory M1 polarization state and inactivating the TLR4/NF-kB pathway reduces inflammatory response attenuates the cognitive disorders from acute limb ischemia-reperfusion.

PNDs are an ordinary postoperative complication among old patients. The release of systemic acute inflammation and proinflammatory cytokines after the operation are critical factors [1, 18]. Peripheral operation and remote organ injury induce neuroinflammation [19], which is a critical factor in cognitive impairment, especially in the hippocampus [20, 21]. Furthermore, circulating monocytes, peripheral systemic elements, neutrophils, macrophages, and the ensuing proinflammatory milieu can play various roles in the central nervous system (CNS), making contributions to variations in neuronal effect and glial homeostasis [22].

Although tourniquets exert a significant effect on vascular or orthopedic operations, some local and integrated complications relate to the application of this equipment, including nerve palsy, hyperdynamic response, and skeletal muscle damage [10, 23]. Prolonged limb tourniquet usage and the follow-up recovery of blood flow leads to inflammation in local ischemic skeletal muscle, moreover, amplification of the inflammation response often results in a complicated cytokine cascade that serves to related secondary remote organs damage [24], and these complications are a risk element for disorders of the CNS. To date, no researchers have paid attention to the association between tourniquet use and cognitive function, so we mimicked the clinical tourniquet-induced critical limb syndrome in the mouse model to verify the correlation. Our findings confirm that the study capacity of mice can be impaired by tourniquet-induced acute limb ischemia-reperfusion, which is accompanied by an increase in inflammatory cytokines including IL-1*β*, TNF-*α* proteins in serum, and NF-kB overexpression in the hippocampus. These results indicate that increased inflammatory cytokines in serum can produce an inflammatory response in the CNS, leading to perioperative neurocognitive disorders.

As innate immune cells of the CNS, microglia are significant participants in the natural immunity and inflammatory response of the CNS [25]. Evidence indicates that the phenotype (M1 and M2) of activated microglia regulates the repair and reproduction response following nerve injury [5, 6]. M1 microglia, labeled CD68, are considered to be proinflammatory and result in deleterious neuroinflammation. For M2 microglia, CD206 as a molecular marker is believed to be anti-inflammatory and to promote regeneration after injury [26, 27]. Our study displayed that activated M1 microglia were grown, as CD68 elevated instead of CD206 in the hippocampus of mice following tourniquet use. Generally, these outcomes mean that microglia in mice with acute limb ischemia-reperfusion might be stimulated toward M1, but not M2.

Toll-like receptors (TLRs) are everywhere in nature that stimulate the inborn immune system in the face of pathogens, stressors, and cytokines [28]. Toll-like receptor 4 (TLR4) is a member of the TLR subtypes, which is stimulated by precipitating element and results in activating nuclear factor kappa B (NF-kB), thereby triggering the transcription of a lot of proinflammatory genes, including prostaglandin E2 (PGE2) and IL-1 [14, 29, 30]. TLR4 is primarily expressed on microglia in the CNS, which could produce proinflammatory cytokines once activated and mediate the neuroinflammatory process [31].

Accumulating research in animal models of many acute inflammations indicates dexamethasone (Dex) has an organ protective function by inhibiting inflammatory cascades [12, 13]. Given the prominent role of Dex on anti-inflammatory, we thus hypothesized that Dex could reduce inflammatory reactions on the CNS following tourniquet use. The study results were concordant with our hypothesis. The learning ability of mice showed impairment after the tourniquet was used, and these changes were ameliorated by Dex preconditioning. This outcome confirms research by Glumac et al. [32], who observed Dex could decrease the risk of early cognitive disorders after cardiac surgery. Moreover, the protein level utterance of TLR-4 and NF-kB in the hippocampus was examined to dig the TLR4 adjusted mechanisms underlying the preprocessing with Dex. Following a past report, it was observed that the utterance of TLR-4 and NF-kB were greatly higher in the *I*/*R* group [14], showing the activation of the TLR-4/NF-kB pathway exerts a significant effect on the pathophysiology of tourniquet-induced *I*/*R* injury. Moreover, the levels of TLR-4 and NF-kB in the hippocampus were significantly lower in mice treated with Dex compared to mice with no treatment. Our results demonstrated that Dex pretreatment suppressed inflammation in the hippocampus and is related to inhibiting the stimulation of TLR-4 and NF-kB. Our results are consistent with previous research results [8].

There are several restrictions in this research. First, the exact mechanism of Dex regulation in tourniquet-induced neurocognitive disorders was not explored. The protective role of Dex may take part in another inflammatory signaling pathway. Second, consider the clinically related concentration and refer to past research [8, 33] 1 mg/kg of Dex intraperitoneal injection in the animal experiment is selected, but different doses of Dex were not tested. Last, the prolonged profits of Dex were not explored, and the long-term roles of this treatment in both local limbs and remote organs observing tourniquet-induced I/R damage were not investigated further.

## 5. Conclusions

The current research shows that the study capacity of mice can be impaired by tourniquet-induced acute inflammation. Pretreatment with Dex, partly through inhibiting microglia transformation to the proinflammatory M1 polarization state and inactivating of the TLR4/NF-kB pathway decreases inflammatory response, attenuates the cognitive disorders in mice following tourniquet used.

## Data Availability

The data used and analyzed during this study are available upon request.
